# The GR of CA1 is involved in anxiety-like behavior induced by 0.8/2.65 GHz dual-frequency electromagnetic radiation

**DOI:** 10.3389/fnmol.2026.1779797

**Published:** 2026-06-22

**Authors:** Bin Sun, Teng Xue, Yani Liu, Sheng He, Mengyao Zhao, Hongmei Zhou, Anning Gao, Xinyu Wang, Yao Ma, Zhihui Li, Guofu Dong, Changzhen Wang

**Affiliations:** 1 Beijing Institute of Radiation Medicine, Beijing, China; 2 The Key Laboratory of Neural and Vascular Biology, Ministry of Education, Department of Biochemistry and Molecular Biology, Hebei Medical University, Shijiazhuang, China; 3 Yangtze University, Jingzhou, China

**Keywords:** anxiety-like behavior, electromagnetic radiation, glucocorticoid receptor, hippocampus, hypothalamic-pituitary-adrenal axis

## Abstract

**Introduction:**

The proliferation of wireless communication has increased environmental exposure to complex radiofrequency electromagnetic radiation (EMR), sparking concerns about its impact on brain function and emotional regulation. Most studies use single-frequency paradigms that do not reflect real-world multifrequency exposure.

**Methods:**

We developed a dual-frequency EMR mouse model (0.8/2.65 GHz, 4 W/kg, 4 h/day for 21 days) to evaluate behavioral, endocrine, neurophysiological, and molecular outcomes.

**Results:**

Dual-frequency EMR induced anxiety-like behaviors without depression-like phenotypes, accompanied by hyperactivation of the hypothalamic–pituitary–adrenal (HPA) axis. Molecular analysis showed selective downregulation of glucocorticoid receptors (GRs) in the hippocampal CA1 region, while mineralocorticoid receptors (MR) and FKBP51 remained unchanged. GR overexpression in CA1 alleviated anxiety-like behavior, whereas GR knockdown exacerbated it and increased corticosterone levels. Effects were consistent in male and female mice, but functional validations were limited to males.

**Discussion:**

Our findings indicate that CA1 GR signaling may mediate the link between dual-frequency EMR exposure, HPA axis dysregulation, and anxiety-like behavior, suggesting GR restoration as a potential therapeutic target for EMR-induced emotional disturbances.

## 1 Introduction

The widespread integration of wireless communication networks has substantially increased daily environmental exposure to EMR. Sources such as mobile phones, Wi-Fi routers, and base stations commonly emit at frequencies including 0.8 and 2.65 GHz (Ahlbom et al., 2008; van Deventer et al., 2011). Although current exposure standards focus primarily on preventing acute thermal damage, emerging evidence suggests that chronic, low-intensity EMR can influence central nervous system function, particularly emotional processing and stress regulation (Eskandani and Zibaii, 2024). Biological effects appear to be frequency dependent (Shehu et al., 2016), yet most experimental models employ single-frequency EMR, which does not accurately represent complex multifrequency exposure in real life (Xue et al., 2024a). Therefore, our study focuses primarily on the potential hazards posed by alternating dual-frequency radiation to living organisms.

The mechanism by which complex-frequency EMR induces affective disturbances remains unclear. In our previous study, we have explored the hypothalamic-pituitary-adrenal (HPA) axis but not in-depth (Sun et al., 2025). Building upon our previous findings, we further explored the molecular mechanism of the HPA axis. Under physiological conditions, the HPA axis regulates glucocorticoid release through a tightly controlled negative feedback loop, contributing to emotional stability and normal anxiety levels (Lee et al., 2024). However, when the body is chronically in an over-activated state of the HPA axis, it can lead to chronic hypercortisolism. High CORT can cause neurotoxicity and neuronal apoptosis by targeting central structures, damaging the functions of brain regions related to emotional regulation such as the hippocampus and amygdala. These structural impairments further exacerbate anxiety-related symptoms (Kiraly et al., 1997; Stokes, 1995; Valsamakis et al., 2019). In previous studies, we found that short-term (last for 7 days) and long-term (last for 28 days) exposure to 2,450 MHz both caused anxiety-like behavior, and is associated with increased CORT (Gupta et al., 2019; Tarsaei et al., 2022). Whereas pharmacological suppression of CORT synthesis mitigates stress-associated anxiety (Novaes et al., 2024). In addition, plasma adrenocorticotropic hormone (ACTH) and CORT levels were enhanced in adult male Wistar rats after prolonged exposure to 900 MHz mobile radiofrequency (6 h daily for 4 weeks) (Shahabi et al., 2018). Therefore, the HPA axis plays a crucial role in emotion regulation in the single-frequency EMR. However, the role of dual-frequency EMR in the mechanism of the HPA axis has rarely been reported.

Through negative feedback regulation, CORT inhibits HPA axis activity, a suppression process critically modulated by the hippocampus (Posener et al., 2001). Abundant GR in the hippocampus—exhibiting low affinity and broad tissue distribution—function as stress-response terminators. When activated by high CORT levels during stress, GR inhibits CRH/ACTH release via negative feedback. Dysfunctional GR disrupts this regulation, causing prolonged CORT secretion and chronic anxiety (Birnstiel et al., 1995; Choi et al., 2008). Furthermore, in mice with forebrain-targeted GR deletion, stress challenge induced significantly increased plasma ACTH and CORT levels concomitant with defective negative feedback control of the HPA axis (Yin et al., 2007). The role of brain GR in modulating emotions is recognized; however, it has not been determined whether dual-frequency EMR modulates GR dynamics in the within limbic circuits involved in emotional regulation.

Through the development of a dual-frequency EMR animal model, this study aims to uncover the functional brain region, molecular mechanisms, and potential therapeutic targets that contribute to anxiety in mice.

## 2 Results

### 2.1 Dual-frequency (0.8/2.65 GHz) electromagnetic radiation exposure provokes anxiety-like behavior in male mice

**FIGURE 1:**
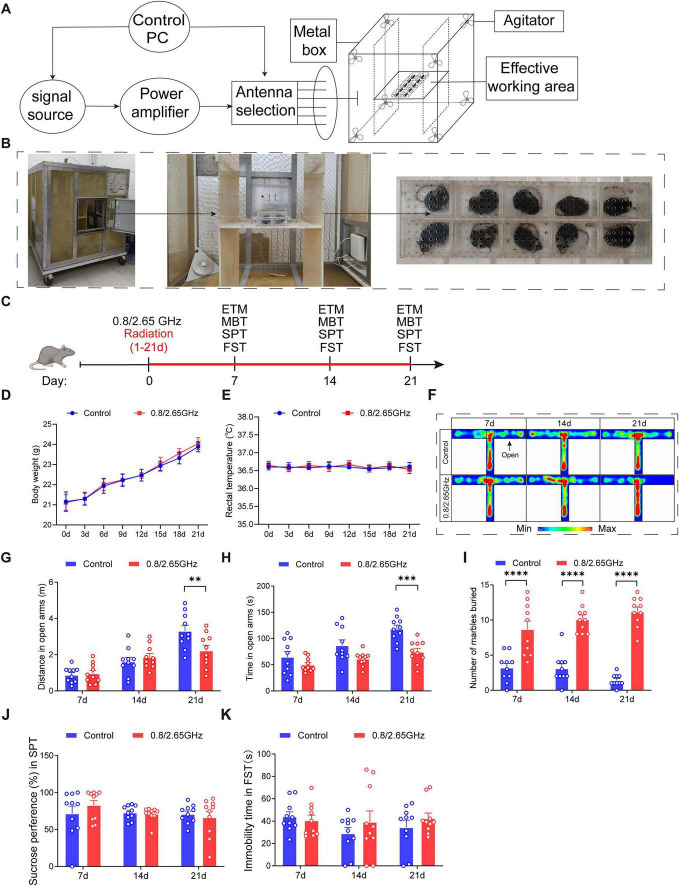
Anxiety-like behavioral evaluation in male mice after exposure to dual-frequency EMR (0.8/2.65 GHz). **(A)** Schematic and **(B)** photograph illustrating the operating principle of the electromagnetic reverberation chamber. **(C)** Experimental schedule: mice were exposed to 0.8 and 2.65 GHz EMR for 2 h each per day (a total of 4 h per day, from 8:00 a.m. to 12:00 p.m.) at 4 W/kg for 21 consecutive days except for days 7, 14, and 21; behavioral tests—including the elevated T-maze (ETM), marble burying test (MBT), sucrose preference test (SPT), and forced swimming test (FST), The same mice group was used for ETM and FST experiments. A separate group was used for the MBT test, and another distinct group for the SPT—were performed on days 7, 14, and 21. Physiological parameters during exposure included **(D)** body weight and **(E)** rectal temperature (*n* = 10/group). **(F–H)** ETM (07:00–09:00, *n* = 10/group): representative heatmaps **(F)**, open-arm distance **(G)**, and time spent in the open arms **(H)**. **(I)** MBT (10:00–12:00, *n* = 10/group): number of marbles buried. **(J)** SPT (5–7, 12–14, 19–21 days of non-irradiation period, *n* = 10/group): sucrose preference (%). **(K)** FST (20:00–22:00, *n* = 10/group): immobility time. Data are presented as mean ± SEM (*n* = 10/group). **p < 0.01, ***p < 0.001, ****p < 0.0001 vs. control. Statistical analyses: repeated-measures ANOVA for **(D,E)** and Two-way ANOVA for **(G–K)**.

Figures 1A,B depict the EMR devices used. Mice were randomly assigned to either a control group or a dual-frequency EMR group (0.8/2.65 GHz). The EMR group received daily 4-h exposures (8:00 a.m. to 12:00 p.m.), consisting of 2 h at 0.8 GHz followed immediately by 2 h at 2.65 GHz, at a whole-body SAR of 4 W/kg. Exposures occurred within the device’s effective working area and were omitted on behavioral testing days (e.g., days 7, 14, 21). Control mice were placed identically but with devices powered off.

Following irradiation, we assessed both physiological indices and behavioral performance (Figure 1C). Body weight and rectal temperature remained comparable between groups throughout the exposure period (Figures 1D,E). In the elevated T-maze (ETM; Figures 1F–H), mice exposed to dual-frequency EMR exhibited reduced exploration of the open arms, with a significant reduction in open-arm distance on day 21 (Figure 1G; *p* = 0.0053) and a significant decrease in open-arm time on day 21 (Figure 1H; *p* = 0.0005) relative to controls. Consistently, the marble burying test (MBT) revealed increased burying activity in the dual-frequency EMR group on days 7, 14, and 21 (Figure 1I; *p* < 0.0001, *p* < 0.0001, and *p* < 0.0001). Together, these findings indicate that exposure to dual-frequency (0.8/2.65 GHz) EMR promotes anxiety-like behavioral phenotypes in mice.

No significant differences were found between the control and dual-frequency (0.8/2.65 GHz) EMR-exposed groups in terms of sucrose preference (SPT) or forced swim test (FST) on days 7, 14, and 21 (Figures 1J,K), leading to the conclusion that this EMR exposure did not trigger depression-like behavior in mice.

### 2.2 Anxiety triggered by dual-frequency EMR was associated with hyperactivation of the hypothalamic–pituitary–adrenal (HPA) axis and diminished neuronal activity in CA1

**FIGURE 2:**
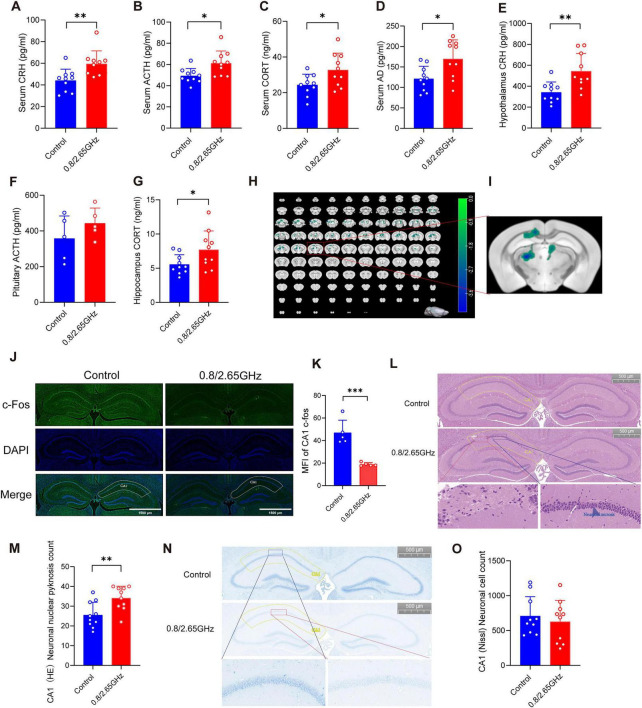
Impact of dual-frequency EMR (0.8/2.65 GHz) on HPA-axis function and CA1 neuronal activity in male mice. **(A–D)** Serum concentrations of CRH, ACTH, CORT, and AD quantified by ELISA (*n* = 10/group). **(E)** Hypothalamic CRH levels measured by ELISA (*n* = 10/group). **(F)** Pituitary ACTH levels determined by ELISA (*n* = 10/group). **(G)** Hippocampal CORT levels assessed by ELISA (*n* = 10/group). **(H,I)** Resting-state fMRI–based regional homogeneity (ReHo) analysis after dual-frequency EMR exposure, showing whole-brain images and a schematic of the CA1 region (*n* = 12/group). **(J)** Representative immunofluorescence images of c-Fos (green), DAPI (blue), and merged channels. **(K)** Quantification of c-Fos mean fluorescence intensity in CA1 (*n* = 5/group). **(L)** Representative schematic of hematoxylin–eosin (HE) staining in CA1. **(M)** Quantification of neuronal nuclear pyknosis in CA1 (*n* = 10/group). **(N)** Representative schematic of Nissl staining in CA1. **(O)** Quantification of Nissl-positive neurons in CA1 (*n* = 10/group). Data are shown as mean ± SEM. *p < 0.05, **p < 0.01, ***p < 0.001; unpaired *t*-test for all comparisons.

**FIGURE 3:**
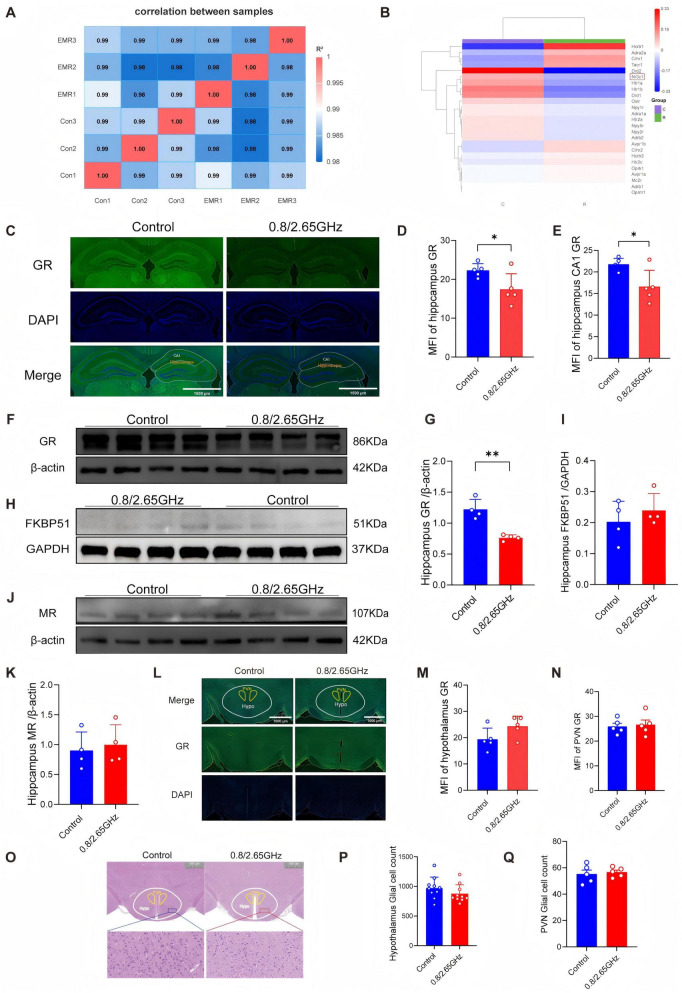
Alterations in GR signaling within CA1 following exposure to 0.8/2.65 GHz dual-frequency electromagnetic radiation. **(A)** Correlation of mouse CA1 region brain tissue. Con: Control; EMR: 0.8/2.65GHz. **(B)** The heat map focused on genes related to the GR pathway, with particular attention to the Nr3c1 gene, which encodes GR protein (*p* < 0.05, *n* = 3 per group). **(C)** Representative immunofluorescent staining illustrating GR distribution, nuclear labeling (DAPI), and merged images in the hippocampus, with emphasis on the CA1 subregion. **(D,E)** Quantification of mean GR fluorescence intensity in the entire hippocampus **(D)** and specifically within CA1 **(E)** (*n* = 5 per group). **(F)** Immunoblot images showing hippocampal GR protein levels with β-actin used as a loading control. **(G)** Densitometric analysis of GR normalized to β-actin in hippocampal tissue (*n* = 4 per group). **(H)** Representative Western blot results for FK506-binding protein 51 (FKBP51) in the hippocampus, with GAPDH as the internal reference. **(I)** Quantitative analysis of FKBP51 expression normalized to GAPDH (*n* = 5 per group). **(J)** Western blot images depicting mineralocorticoid receptor (MR) expression in the hippocampus, with β-actin serving as the loading control. **(K)** Statistical evaluation of MR/β-actin protein ratios (*n* = 5 per group). **(L)** Representative immunofluorescence images of GR and DAPI staining in the hypothalamus. **(M)** Mean GR fluorescence intensity measured in the hypothalamus (*n* = 5 per group). **(N)** Mean GR fluorescence intensity measured in the paraventricular nucleus of the hypothalamus (*n* = 5 per group). **(O)** Schematic representation of hematoxylin–eosin (HE) staining in hypothalamic sections. **(P)** Quantification of glial cell numbers in the hypothalamus (*n* = 10 per group). **(Q)** Quantification of glial cell numbers in the paraventricular nucleus of the hypothalamus (*n* = 10 per group). Data are presented as mean ± SEM. Statistical significance was determined using an unpaired *t*-test. *p < 0.05, **p < 0.01.

A large number of studies have shown that there are abnormal changes in the HPA axis function of patients with anxiety disorders. Based on this, we first tested the HPA axis hormone content, found that serum corticotropin releasing hormone (CRH), adrenocorticotropic hormone (ACTH) and Corticosterone (CORT) significantly increase (*p* = 0.0066, Figure 2A; 0.0123, Figure 2B; 0.0297; Figure 2C), indicating that the HPA axis is overactive in the radiation group compared to the control group. Similarly, we also examined Adrenaline (AD) in the serum, finding that a significant increase in the serum exposed to dual-frequency EMR compared to the control group (*p* = 0.0129, Figure 2D), is associated with anxiety-like behavior. Furthermore, we also investigated the HPA axis in the central system (Figures 2E–G), found that whole Hypothalamus CRH and Hippocampus CORT significantly increase (*p* = 0.0042, Figure 2E; *p* = 0.0422; Figure 3G), suggesting that the HPA axis in the central system is also involved in the anxiety-like behavior.

To further identify the specific brain regions involved in anxiety induction in mice, we conducted a series of investigations. Using functional magnetic resonance imaging (fMRI), we examined changes in neural activity induced by dual-frequency EMR. The results revealed that, compared to control mice, the irradiated group showed a significant reduction in regional homogeneity (ReHo) within the CA1 region, reflecting poorer synchronization between local and adjacent voxels (*p* < 0.05, Figures 2H,I).

Additionally, several other nuclei exhibited notable decreases in ReHo, though these are not strongly linked to anxiety. These regions include:

(1) the anterior pretectal nucleus (APT), which is primarily implicated in memory processes (Kim et al., 2020);

(2) the paraventricular thalamic nucleus (PVT), known for its key roles in arousal, pain perception, and integration of sensory information, serving as a critical hub within functional brain networks (Ren et al., 2018);

(3) lobule III of the cerebellum, which is mainly involved in regulating muscle tone and motor control of trunk and proximal limb muscles according to cerebellar functional organization (Hamre and Goldowitz, 1997);

(4) crus II of the ansiform lobe (Crus2), a region associated predominantly with cognitive functions and motor coordination (Zhai et al., 2024).

Subsequently, immunofluorescence analysis (Figures 2J,K) revealed a marked reduction in c-Fos signal intensity (*p* = 0.0005, Figure 2K), suggesting decreased neuronal activation. Furthermore, chronic overactivation of the hypothalamic–pituitary–adrenal (HPA) axis can lead to persistently elevated corticosterone (CORT) levels, which may induce neurotoxicity, promote neuronal apoptosis, and impair the functionality of emotion-related brain regions such as the hippocampus and hypothalamus. To evaluate whether these effects were accompanied by structural alterations, we performed Hematoxylin and Eosin (HE) staining and Nissl staining on the hippocampal CA1 region (Figures 2L–O). The results showed a significant increase in the number of neurons with nuclear pyknosis in irradiated mice compared with controls (*p* = 0.0067, Figure 2M), which may indicate more severe neurons with nuclear pyknosis, supporting the presence of neuronal damage. Collectively, these findings indicate that reduced neuronal activity within the CA1 region contributes to anxiety-like behaviors induced by dual-frequency EMR.

In summary, our results suggest that peripheral and central overactivation of the HPA axis, coupled with diminished CA1 activity, disrupts physiological homeostasis and ultimately leads to the emergence of anxiety-like behavior in mice.

### 2.3 Dual-frequency (0.8/2.65 GHz) EMR significantly lowered GR expression in mouse cornu ammonis (CA1)

Glucocorticoid-mediated negative feedback regulation of the HPA axis plays a key role in neuropsychiatric disorders. Studies using the chronic restraint stress (CRS) model have shown that reduced GR expression in the prefrontal cortex may contribute to anxiety- and depression-like behaviors (Chiba et al., 2012). However, in the context of EMR exposure, it remains unclear whether GR participates in the anxiety-like behavior observed in mice.

In order to further clarify the relevant mechanisms, we performed transcriptome sequencing in the CA1 region (Figures 3A,B), and cluster analysis revealed a significant downregulation of the Nr3c1 gene in the GR pathway following irradiation (*p* = 0.0355, Figure 3B). Then, immunofluorescence experiments were performed (Figures 3C–E), we found the GR in the hippocampus significantly decreased (*p* = 0.0379, Figure 3D), further the GR in the CA1 region also decreased (*p* = 0.0316, Figure 3E) in the dual-frequency EMR group compared to the control group. The expression of GR in the RNK group showed a decreasing trend compared to the RN group (with no significant difference), suggesting that it plays a certain regulatory role in exacerbating behavioral disorders. Western blot (WB) detection of hippocampal tissue revealed a significant downregulation of GR expression in the radiation group relative to the control group (*p* = 0.0016, Figures 3F,G), demonstrating that prolonged dual-frequency EMR exposure impacted GR levels in this brain region. FKBP51 can bind to GR, keeping GR in an inactive state and reducing its sensitivity to cortisol, thereby weakening the negative feedback regulation of the HPA axis and leading to an increase in cortisol levels. However, our investigations found that FK506 binding protein 51 (FKBP51) that binding GR had no significant difference (Figures 3H,I). Next, we also detected the hippocampus Mineralocorticoid receptors (MR) content. Under physiological conditions, the MR distributed in the hippocampus have a high affinity for corticosterone. They can be activated in the basal state and participate in maintaining homeostasis and governing the initial stress response. However, there are no significant differences between the control and radiation group (Figures 3J,K). Lastly, we also detected the GR of the whole hypothalamic region and PVN (Figures 3L,N) and found that there was no significant difference, and the glial cell count of the hypothalamus and PVN also showed no significant difference (Figures 3O–Q). GR expression and glial cell counts in the hypothalamus and PVN did not show significant alterations under the present experimental conditions. In summary, these results suggest that the downregulation of GR in the hippocampus, especially in the CA1, is involved in anxiety-like behavior in mice.

### 2.4 GR overexpression in the CA1 region mitigated anxiety-like behavior resulting from exposure to dual-frequency (0.8/2.65 GHz) EMR

**FIGURE 4:**
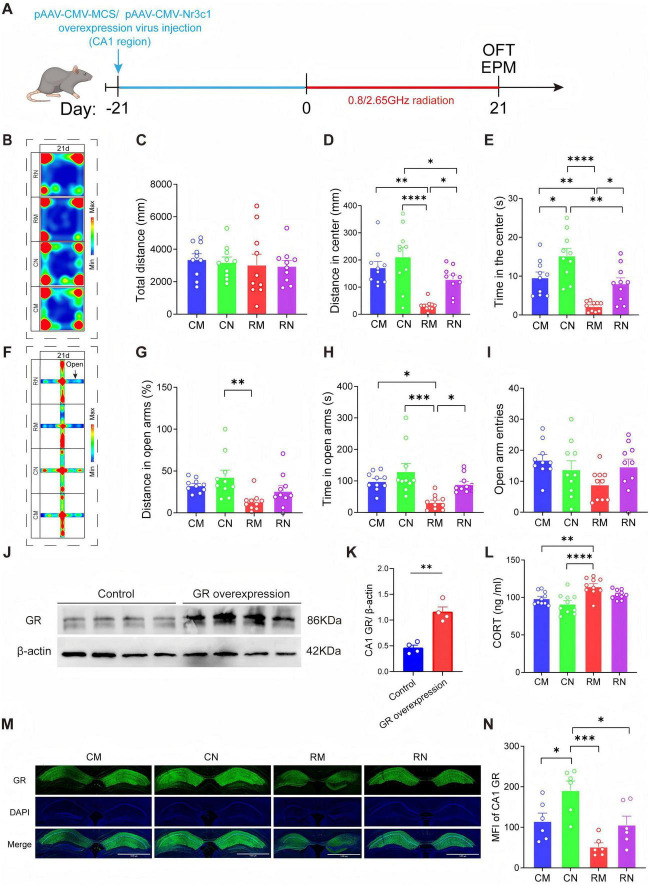
GR overexpression in CA1 alleviates dual-frequency EMR-induced anxiety in mice. **(A)** Radiation schedule (4 W/kg, 21 days) with group-specific time blocks for RM and RN (RM group: 10:00–12:00, 0.8 GHz; 12:00–14:00, 2.65 GHz; RN group: 14:00–16:00, 0.8 GHz; 16:00–18:00, 2.65 GHz). Behavioral assessments: **(B–E)** OFT results (day 21, 8:00–12:00) including heatmaps **(B)**, total distance **(C)**, center distance **(D)**, and center time **(E)**. **(F–I)** EPM results (day 21, 18:00–22:00) including heatmaps **(F)**, open arm distance % **(G)**, open arm time **(H)**, and open arm entries **(I)**. **(J)** Western blot results of GR protein in CA1 region, with β-actin as internal reference. **(K)** Quantitative analysis of GR expression, normalized to β-actin (*n* = 4 per group). **(L)** Immunofluorescence for GR and DAPI in CA1. **(M)** Quantified GR fluorescence intensity in CA1. **(N)** Serum CORT levels by ELISA. All panels: *n* = 10/group, except K where *n* = 6. Data are mean ± SEM. Significance (*p < 0.05, **p < 0.01, ***p < 0.001, ****p < 0.0001) was determined by one-way ANOVA. Groups: CM (Control + MCS), CN (Control+Nr3c1), RM (Radiation + MCS), RN (Radiation+Nr3c1).

Based on the critical involvement of the GR in emotion regulation and the alterations in GR activity observed in the CA1 region of mice following dual-frequency EMR exposure in this study, we next sought to examine if enhancing GR function in this region could counteract the resulting neurobehavioral deficits. To do so, we injected either an Nr3c1-overexpression virus (pAAV-CMV-Nr3c1-3 × FLAG-P2A-mCherry-tWPA) or an MCS control virus (pAAV-CMV-MCS-mCherry-tWPA) into the CA1; after 21 days, EMR exposure was administered and behavioral tests were performed (Figure 4A).

The results showed that (Figures 4B–E), in the OFT, compared with the RM group (Radiation 0.8/2.65 GHz + MCS), the RN group (Radiation 0.8/2.65 GHz + Nr3c1 Overexpression) exhibited a significant increase in both center distance (*p* = 0.0441; Figure 4D) and time spent in the center (*p* = 0.0259; Figure 4E) on day 21. Similarly, in the EPM test (Figures 4F–I), the RN group spent significantly more time in the open arms than the RM group (*p* = 0.0349; Figure 4H). Twenty-one days after injection of the overexpression virus into the hippocampal CA1 region, western blot (WB) analysis revealed significantly higher GR protein expression in the overexpression group compared to the sham surgery group (*p* = 0.0020; Figures 4J,K). Furthermore, serum hormone analysis showed a non-significant decreasing trend in CORT levels in the RN group relative to the RM group (*p* = 0.1096; Figure 4L). Although not statistically significant, a trend toward higher GR expression was also observed in the RN group compared to the RM group (*p* = 0.2176; Figures 4M,N). The results showed that GR overexpression in the CA1 region modestly alleviated anxiety-like behaviors induced by dual-frequency EMR.

### 2.5 GR knockdown in the CA1 region aggravated anxiety-like behavior induced by exposure to dual-frequency (0.8/2.65 GHz) EMR

**FIGURE 5:**
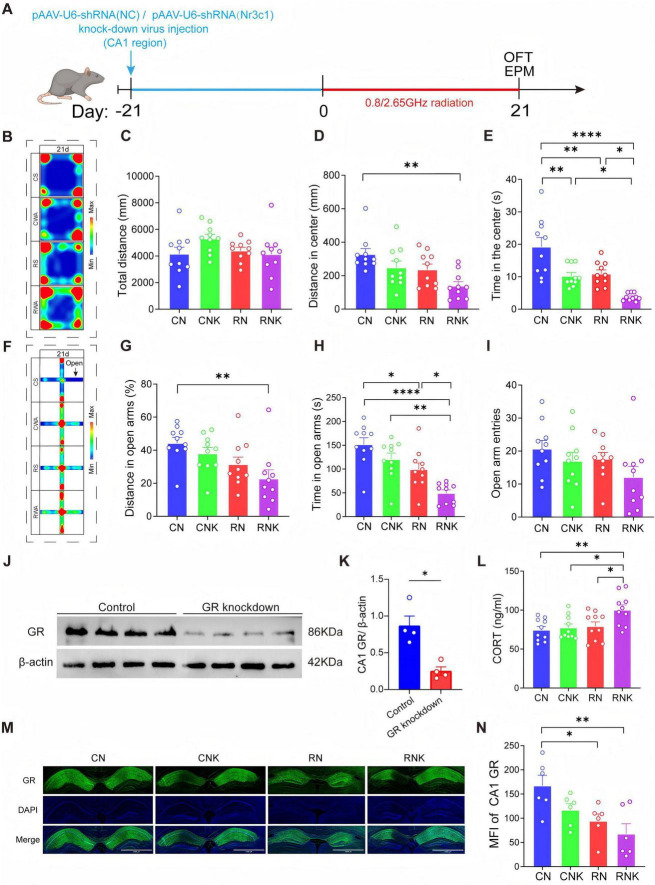
Silencing GR expression in hippocampal CA1 neurons intensifies anxiety-like behaviors following electromagnetic radiation exposure. **(A)** Experimental timeline illustrating the dual-frequency electromagnetic radiation protocol (4 W/kg, 21 consecutive days). Animals in the RN group were exposed to 0.8 GHz radiation from 10:00–12:00 and 2.65 GHz from 12:00–14:00, whereas the RNK group received 0.8 GHz radiation from 14:00–16:00 and 2.65 GHz from 16:00–18:00. **(B–E)** Open field test (OFT) performed on days 7, 14, and 21 (8:00–12:00). Representative locomotion heat maps at day 21 (*n* = 10 per group) are shown in **(B)**. Quantitative analyses include total travel distance **(C)**, distance covered in the central zone **(D)**, and duration spent in the center area **(E)**. **(F–I)** Elevated plus maze (EPM) assessment conducted on day 21 (18:00–22:00). Representative trajectory heat maps are presented in **(F)** (*n* = 10 per group). Behavioral parameters analyzed include the percentage of distance traveled in open arms **(G)**, time spent in open arms **(H)**, and the frequency of open-arm entries **(I)**. **(J)** Western blot results of GR protein in CA1 region, with β-actin as internal reference. **(K)** Quantitative analysis of GR expression, normalized to β-actin (*n* = 4 per group). **(L)** Serum corticosterone (CORT) concentrations measured using ELISA (*n* = 10 per group). **(M,N)** Immunofluorescent staining of GR in the hippocampal CA1 region. Representative images of GR, DAPI, and merged channels are shown in **(M)**, with corresponding quantitative analysis of mean GR fluorescence intensity in CA1 neurons (*n* = 6 per group) shown in **(N)**. Data are presented as mean ± SEM. Statistical significance was determined by one-way ANOVA. *p < 0.05, **p < 0.01, ****p < 0.0001. Experimental groups: CN, control + negative control; CNK, control + Nr3c1 knockdown; RN, radiation (0.8/2.65 GHz) + negative control; RNK, radiation (0.8/2.65 GHz) + Nr3c1 knockdown.

Since our results show that CA1-specific GR overexpression alleviates anxiety-like behaviors in mice, it is important to determine whether reducing GR in CA1 produces the opposite effect and exacerbates anxiety-like phenotypes. Mice received bilateral CA1 injections of either an Nr3c1 knockdown virus (pAAV-U6-shRNA (Nr3c1)-CMV-EGFP-WPRE) or a NC control virus (pAAV-U6-shRNA (NC)-CMV-EGFP-WPRE). Twenty-one days later, EMR modeling was performed followed by behavioral assessments (Figure 5A).

The results demonstrated that in the open field test (OFT; Figures 5B–E), the RNK group (Radiation 0.8/2.65 GHz + Nr3c1 knockdown) spent significantly less time in the center compared to the RN group (Radiation 0.8/2.65 GHz + NC) on day 21 (*p* = 0.0189; Figure 5E). Similarly, in the elevated plus maze (EPM; Figures 5F–I), the open arm duration was markedly shorter in the RNK group relative to the RN group on day 21 (*p* = 0.0283; Figure 5H). Twenty-one days after injection of knockdown virus into the hippocampal CA1 region, western blot (WB) analysis revealed significantly reduced GR protein expression in the knockdown group compared to the sham surgery group (*p* = 0.0125, Figures 4J,K). Serum CORT levels were significantly elevated in the RNK group compared to the RN group (*p* = 0.0423; Figure 5L), suggesting that CA1-specific GR knockdown enhances the perturbation of CORT levels induced by dual-frequency EMR. Analysis of GR expression (Figures 5M,N) revealed a downward trend between the RNK and RN groups. These findings indicate that knockdown of Nr3c1 in the CA1 region amplified anxiety-like behaviors and the dysregulation of the HPA axis induced by dual-frequency EMR.

### 2.6 Validation of GR alterations in female mice following dual-frequency EMR exposure

**FIGURE 6:**
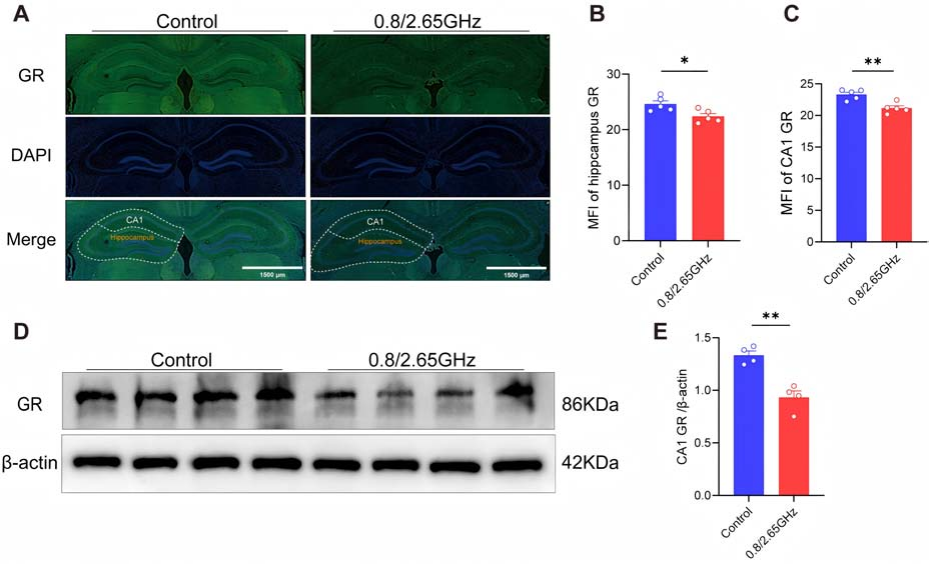
Validation of hippocampal and CA1 glucocorticoid receptor (GR) alterations in female mice following dual-frequency (0.8/2.65 GHz) electromagnetic radiation exposure. **(A)** Representative immunofluorescence images showing GR expression in the hippocampus of female mice after dual-frequency EMR exposure. **(B)** Quantification of hippocampal GR fluorescence intensity in female mice. **(C)** Quantification of GR fluorescence intensity in the CA1 region of female mice. **(D)** Representative western blot images showing GR protein expression in the hippocampal CA1 region of female mice. **(E)** Quantification of GR protein levels normalized to β-actin in the CA1 region. Data are presented as mean ± SEM. Statistical analyses were performed using unpaired Student’s *t*-test. *p < 0.05, **p < 0.01 versus control group.

To explore whether sex differences influence HPA axis regulation, we performed validation experiments in female mice. Immunofluorescence showed that dual-frequency electromagnetic radiation (EMR) significantly reduced GR expression in the whole hippocampus (*p* = 0.0212, Figures 6A,B) and specifically in the CA1 region (*p* = 0.0031, Figure 6C). Western blot analysis confirmed that GR protein levels in the CA1 region were markedly downregulated following EMR exposure (*p* = 0.0027, Figures 6D,E). Together with previous findings in males, these results indicate that EMR-induced suppression of hippocampal CA1 GR expression occurs consistently across both sexes. However, whether this molecular downregulation directly contributes to anxiety-like behavior in females, or can be reversed by Nr3c1 overexpression, remains to be determined, as viral and behavioral manipulations in the present study were restricted to male mice.

## 3 Discussion

Dual-frequency EMR exposure was found to elicit anxiety-related behavioral changes in mice, accompanied by pronounced alterations in the HPA axis across peripheral blood and central nervous structures. A significant decrease in glucocorticoid receptor activity was observed in the hippocampal CA1 region (including male and female mice), indicating a disruption in stress hormone signaling. Functional MRI analysis revealed diminished ReHo values in CA1, pointing to impaired local network synchronization. Consistent with imaging data, biochemical analyses confirmed reduced GR expression in hippocampal tissue. Experimentally increasing CA1 GR levels attenuated EMR-related anxiety, whereas GR suppression produced the opposite effect. These findings collectively suggest that CA1 GR signaling is associated with, rather than definitively an associated mechanism in the neurobiological response to EMR and may represent a potential therapeutic target.

Our findings demonstrate that chronic dual-frequency (0.8/2.65 GHz) EMR exposure induces significant anxiety-like behaviors in mice without eliciting depression-like phenotypes. These results are generally consistent with previous reports showing that single-frequency electromagnetic radiation can influence emotional behaviors (Tarsaei et al., 2022). Notably, anxiety-like behaviors became progressively more pronounced over time and were most evident after 21 days of exposure, whereas earlier time points showed comparatively weaker behavioral alterations. Based on these findings, subsequent molecular and mechanistic analyses were focused on the 21-day time point, which best reflected stable anxiety-like phenotypes. This time-dependent pattern is also supported by previous studies indicating that short-term RF-EMR exposure may induce only transient or adaptive responses, whereas prolonged exposure paradigms of at least 3–4 weeks are more likely to produce persistent neurobehavioral abnormalities. Together, these observations suggest that chronic rather than acute EMR exposure plays a critical role in the development of anxiety-like behaviors.

The HPA axis plays a crucial role in both the development and manifestation of anxiety-like behaviors, involving both central and peripheral mechanisms. Chronic overexpression of CRH in the central amygdala has been shown to induce HPA axis hyperactivity, significantly increasing anxiety-like behaviors in the elevated plus maze (EPM) test, as indicated by reduced time spent exploring the open arms (Kalin, 2018). Similarly, in the peripheral system, the fermented black bean preparation Semen Sojae Praeparatum (SSP) ameliorated anxiety symptoms in mice by suppressing HPA axis overactivity. At the human level, dysregulated HPA-axis activity has been repeatedly associated with anxiety vulnerability and onset, including evidence that elevated cortisol awakening response predicts first-onset anxiety disorders (Adam et al., 2014). However, studies investigating the effects of dual-frequency EMR on HPA axis function in the context of anxiety remain limited. In the study, we found that HPA axis in the peripheral and central system both is involved in the anxiety-like behavior, and the content of hormone all are increased. However, given that our measurements were taken only at a single time point (21 days post-irradiation), these findings are subject to temporal constraints. While the results suggest that anxiety-like behaviors observed following EMR exposure are associated with excessive HPA axis activation, they do not establish it as the sole causative factor.

The GR is a key mediator of the physiological stress response and plays an essential role in regulating emotion (Gjerstad et al., 2018). It is most abundantly expressed in limbic structures, including the hippocampus, prefrontal cortex, and amygdala (Murani et al., 2025). Its primary role involves mediating negative feedback regulation of the hypothalamic-pituitary-adrenal (HPA) axis (Gjerstad et al., 2018; Jankord and Herman, 2008; Liu et al., 2023). In our previous study, compared with control mice, the irradiated group exhibited a significant reduction in regional homogeneity (ReHo) within the CA1 region, reflecting poorer synchronization between local and adjacent voxels. This indicates weaker coordination of local neural activity, which was further supported by a significant decrease in neuronal activity in CA1 as shown by c-Fos expression. We subsequently investigated whether GR expression in the CA1 region was altered. Immunofluorescence (IF) and Western blot (WB) analyses revealed a significant decrease in glucocorticoid receptor (GR) levels in the hippocampus, particularly within the CA1 region—a subarea implicated in anxiety regulation (Jia et al., 2022; Li et al., 2023; Ma et al., 2024)—in both male and female subjects. We hypothesize that the reduction in GR impairs the negative feedback mechanism of the HPA axis, leading to its sustained hyperactivity and ultimately resulting in anxiety-like behaviors. These findings suggest that the CA1 region may plays a critical role in mediating anxiety induced by dual-frequency EMR in mice. Through targeted manipulation of GR expression in the CA1 region via both overexpression and knockdown, we demonstrated that GR overexpression alleviated anxiety-like behaviors, whereas GR knockdown exacerbated them. In contrast, while these behavioral outcomes were robust, the corresponding changes in endocrine and protein levels under EMR conditions did not consistently reach statistical significance, warrting a cautious interpretation. It is worth noting that our recent work demonstrated that this exact dual-frequency EMR protocol also induces baseline anxiety-like phenotypes in female mice (Sun et al., 2025). However, the downstream causal role of the hippocampal CA1 GR pathway in driving or mitigating these emotional deficits was investigated here exclusively in males. Future studies employing parallel viral interventions in female cohorts will therefore be important for clarifying sex-specific mechanistic interactions. It has been reported that under stress, expression of FK506 binding protein 51 (FKBP51) increases, which further inhibits GR activity and predisposes the HPA axis to excessive activation (Hartmann et al., 2021). However, in our study, no significant change in FKBP51 was observed. Additionally, the mineralocorticoid receptor (MR), which is constitutively active in the hippocampus, has a high affinity for corticosterone and plays a major role in the initial stress response (Johnson et al., 2024). We did not observe any significant changes in MR following dual-frequency EMR exposure. In summary, our results support the view that modulation of GR may represent a potential strategy for alleviating anxiety-like behavior associated with dual-frequency EMR exposure. Furthermore, we observed a significant increase in the number of necrotic neurons in the CA1 region after dual-frequency EMR exposure. We propose that dual-frequency EMR induces HPA axis hyperactivity and elevates corticosterone levels. Sustained hypercortisolism may, in turn, exert neurotoxic effects on CA1 neurons and impair synaptic plasticity—a mechanism similar to that observed in major depressive disorder (MDD) and post-traumatic stress disorder (PTSD).

In conclusion, prolonged exposure to dual-frequency EMR led to excessive activation of the HPA axis and continuously elevated corticosterone levels in mice. These findings suggest that alterations in CA1 GR signaling may contribute to impaired HPA-axis feedback and the development of anxiety-like behavior, although direct causality remains to be further established. This resulted in downregulation of GR in the CA1, which significantly impaired the negative feedback mechanism of the HPA axis. Consequently, cortisol accumulated further, causing damage to hippocampal neurons and ultimately contributing to the development of anxiety-like disorders.

However, several limitations of the present study should be acknowledged. First, the current exposure paradigm involved sequential rather than truly simultaneous multifrequency EMR stimulation, as this design was selected to ensure dosimetric stability and reproducibility, while fully concurrent multifrequency exposure remains technically challenging. Second, although rectal temperature monitoring did not reveal significant systemic heating, localized microthermal effects in deep brain regions such as the hippocampus cannot be completely excluded. In addition, our mechanistic investigation mainly focused on HPA axis hyperactivation and GR-related alterations without further exploring neural circuit involvement. Finally, although anxiety-like behaviors were observed in mice, species differences and heterogeneous human exposure patterns limit the direct translational relevance of the current findings, and future clinical or epidemiological studies are still needed.

In future studies, thermal and putative non-thermal effects should be further distinguished through rigorous temperature monitoring and standardized RF dosimetry procedures (Foster and Glaser, 2007). In addition, we plan to further investigate the neural circuit mechanisms underlying EMR-induced anxiety by modulating hippocampal CA1 neuronal activity and examining its connectivity with other brain regions, while also exploring CA1 GR-targeted interventions as potential therapeutic strategies for anxiety-like behaviors.

## 4 Materials and methods

### 4.1 Animals

Male and female C57BL/6J mice aged 8 weeks (20.00 ± 0.43 g) were purchased from SPF Biotechnology Co., Ltd. (Beijing, China). Animals were housed under standard conditions on a 12-h light/12-h dark schedule, with lights on from 07:30 to 19:30, room temperature of 22 ± 2°C, and 50–60% relative humidity. Mice had *ad libitum* access to regular chow and tap water for the duration of the experiments. All protocols were reviewed and approved by the Animal Ethics and Protection Committee of the National Beijing Center for Drug Safety Evaluation and Research (Approval No. IACUC-DWZX-2024-595). To minimize experimental bias, all behavioral assessments were performed by researchers blinded to the group allocation (control vs. dual-frequency EMR exposure). The personnel responsible for EMR exposure (modeling) were distinct from those conducting behavioral tests.

### 4.2 EMR exposure equipment

The electromagnetic reverberation chamber (RC) used in this work was developed by Wu Tongning’s group at the Department of Environment and Security, China Academy of Information and Communications Technology (Li et al., 2016). It is a large reinforced-concrete, shielded facility equipped with highly conductive reflective inner surfaces and multiple mechanical mode stirrers. These stirrers, whose continuous rotation alters the chamber’s boundary conditions, enable the generation of a statistically uniform, isotropic, and randomly polarized electromagnetic environment. Key components including signal generators, power amplifiers, and shielding structures are integrated within the system, which is capable of generating electromagnetic waves across a 0–3 GHz frequency spectrum. For this experiment, frequencies of 0.8 and 2.65 GHz were applied at 4 W/kg, with electric field intensity calculated based on murine average body weight according to the parameters specified in Table 1. The RF-EMF exposure paradigm (0.8 GHz for 2 h + 2.65 GHz for 2 h per day, whole-body SAR = 4 W/kg, for 3 weeks) was designed with both environmental relevance and thermal control in mind. The selected carrier frequencies map onto widely deployed mobile communication bands, with ∼800 MHz used in cellular services and ∼2.6 GHz covered by standardized 3GPP operating bands, thereby improving the ecological representativeness of the exposure scenario (800 MHz Cellular Service | Federal Communications Commission, n.d.). Dual-frequency exposure was chosen to better approximate real-world mixed-signal conditions, and the same dual-frequency 2.65/0.8 GHz, SAR 4 W/kg, 4 h/day for 21 days protocol has been reported in mice, where rectal temperature increased by < 1 °C, enabling evaluation of biological outcomes under conditions that minimize confounding thermal load (Xue et al., 2024b). Importantly, the SAR level was selected at the high end commonly used in experimental studies but interpreted in the context of established RF dosimetry and safety guidance, which emphasizes that whole-body RF effects are primarily mediated by temperature elevation; therefore, monitoring/maintaining minimal temperature rise supports a focus on non-thermal mechanisms (Foster and Glaser, 2007; International Commission on Non-Ionizing Radiation Protection [ICNIRP], 2020).

**TABLE 1:** Electric field strength values calculated for mice of different body weights under multiple frequency conditions.

Weight of mice (g)	Radiation frequency (GHz)	Electric field intensity (V/m)
21.9	2.65	188.93
25.0	2.65	189.79
30.0	2.65	190.00
21.9	0.80	243.00
25.0	0.80	262.00
30.0	0.80	285.00

### 4.3 ETM

The apparatus, manufactured by Shanghai Xinruan Technology Co., Ltd. (Shanghai, China), is an elevated T-maze (ETM), similar to the high-Loft Cross maze, forms a “T” shape with one closed arm and two open arms. Both open and closed arms of the mouse ETM measure 30 cm in length and 5 cm in width, while the closed arm reaches 15 cm in height. The central platform measures 5 cm in both length and width, positioned 40 cm above the ground. Prior to the experiment, mice were acclimated in the experimental environment for over 30 min. During testing, they were immediately placed on the central platform of the ETM with their heads facing a fixed open arm. A camera recorded the animals’ movement trajectories for 5 min. Statistical analysis focused on the distance traveled, dwell time, and entry frequency into the open arm across different groups. Significantly, high-anxiety mice exhibited markedly shorter distances and reduced dwell time in the open arm compared to low-anxiety counterparts. Behavioral assessments were carried out in a calm, quiet room. After each trial, the apparatus was cleaned with alcohol to minimize contamination and reduce potential carryover effects.

### 4.4 MBT

This experimental method (Marble Burying Test, MBT) evaluates anxiety-like behaviors and compulsive behaviors in mice. Conducted under dim lighting, the test uses a polypropylene cage (42 × 24 × 12 cm) with a metal mesh top, filled with 5 cm of mouse corn cobs bedding. Fifteen 1.5 cm diameter glass marbles are evenly distributed across the bedding. Two days before testing, two marbles are placed in the cage to prevent new phobias. On test day, mice first explore an empty cage for 30 min to adapt. Then, 15 marbles were evenly distributed in the form of “3 × 5” in the test cage, where the mice remain exposed to them for 30 min while being videotaped. After testing, at least two-thirds of the marbles are counted buried under the bedding. The burying behavior shows a positive correlation with anxiety levels.

### 4.5 SPT

This sucrose preference test (SPT) evaluates the depressive-like behavior in mice. Prior to testing, mice were housed individually in cages for environmental acclimation. During the 48-h training period, two water bottles (1% sucrose solution and purified water) were provided simultaneously to familiarize the mice with the dual-bottle drinking pattern. The positions of the sugar and water bottles were rotated every 12 h during this phase. After a 12-h water deprivation period, the mice entered the test phase (24 h). The specific schedule was as follows: At 12:00 a.m. on days 5, 12, and 19 of the modeling period, the adaptation phase for each sugar water experiment began. On days 5, 12, and 19 at midnight, and on days 6, 13, and 20 at 12:00 a.m., as well as at midnight on days 6, 13, and 20, the sugar water and water bottles were switched. The formal sugar water experiment was conducted 24 h after the final sugar water bottle exchange during the adaptation phase. From 8:00 a.m. to 12:00 p.m. during the adaptation phase, the mice were removed from the sugar water adaptation cage and subjected to a normal electromagnetic radiation experiment. In the formal experiment phase, since no radiation was required, the mice underwent a normal 24-h period before the sugar water experiment. During this phase, one bottle contained 1% sucrose solution while the other remained pure water. The weight of both bottles was recorded before and after the training period. The consumption amounts of sugar and water were calculated by subtracting the post-training bottle weight from the pre-training bottle weight (g), and the preference rate was determined using the formula: Sugar preference (%) = [Sugar Consumption / (Sugar Consumption + Water Consumption)] × 100%. Bottle nozzles were cleaned daily to prevent odor contamination. Both control and radiation groups underwent testing under identical conditions to eliminate environmental factors. A significantly reduced preference rate indicates increased depressive-like symptoms in the mice.

### 4.6 FST

The forced swim apparatus, procured from Anhui Zhenghua Biological Instrument Equipment Co., Ltd. (Anhui, China), consisted of a cylindrical chamber (10 cm diameter × 25 cm height) filled with warm water maintained at 23–25°C. Prior to testing, water depth was adjusted to 15 cm to prevent tail contact with the bottom. Following a 2-h environmental acclimation period, each mouse was placed into the chamber, where 6-min video recording was initiated immediately using specialized software. During this session, the initial 2 min were designated as adaptation, while immobility time during the final 4 min was quantified to assess depression-like behavior. Before the experiment, the mouse should be allowed to become accustomed to the testing environment for a minimum of 2 h. Throughout all trials, auditory stimuli were minimized, and water was replaced between subjects to ensure hygienic consistency.

### 4.7 OFT

The open field test (OFT) apparatus, obtained from Shanghai Xinruan Technology Co., Ltd., consisted of a 50 × 50 × 40 cm^3^ polypropylene arena in which each mouse was placed in a designated corner for 5-min free exploration, with spontaneous activity recorded and analyzed via specialized software. Anxiety-like behaviors were evaluated using key index—including total distance traveled, central zone distance, and central zone duration—following a minimum 2-h environmental acclimation period.

### 4.8 EPM

The elevated plus maze apparatus, acquired from Anhui Zhenghua Biological Instrument Equipment Co., Ltd. (Anhui, China), was constructed with a 50 cm central platform and four arms (50 × 5 cm)—two open and two enclosed by 15 cm walls. During 5-min trials, mouse movements were recorded by an overhead camera and analyzed via specialized software, with anxiety-like behaviors evaluated using key parameters including open-arm distance traveled, open-arm duration, and open-arm entries. To minimize stress, mice were acclimated to the testing environment for at least 2 h prior to experimentation, while auditory stimuli were minimized throughout procedures. Between trials, the maze was thoroughly cleaned with 75% ethanol to eliminate residual odor interference.

### 4.9 Functional magnetic resonance imaging

For specific operations, please refer to the previously published paper (Sun et al., 2025). Data preprocessing comprised slice-timing correction, despiking, motion correction, and nuisance regression with 24 head motion parameters. Functional images were coregistered to individual anatomical scans and normalized to the Turone Mouse Brain Template and Atlas (TMBTA), before applying a 0.01–0.1 Hz temporal filter. Regional homogeneity (ReHo) maps were generated, and intergroup differences were assessed using two-sample *t*-tests (*p* < 0.05, cluster size ≥ 10 voxels) with AFNI’ s 3dttest++. EMR-exposed groups by Shanghai Ji Ying Technology Co., Ltd. ReHo is a commonly used resting-state fMRI (rs-fMRI) index that quantifies the temporal consistency of local neural activity within a brain area. Specifically, it is calculated using Kendall’s coefficient of concordance (KCC) to measure agreement between the time series of each voxel and those of its 26 nearest neighboring voxels. Higher ReHo values indicate stronger local neural activity coordination.

### 4.10 Immunofluorescence

From 22:00 to 24:00 on the 21st day, the mice were anesthetized with 0.7% sodium pentobarbital, and their limbs were secured. A surgical thoracotomy was performed to expose the heart for perfusion. During perfusion, saline was injected into the left ventricle until systemic pallor was observed, followed by immediate replacement with 4°C 4% paraformaldehyde for continued perfusion until limb rigidity occurred. After decapitation, the whole brain was carefully extracted and fixed overnight in 4% paraformaldehyde. Subsequently, the tissue underwent cryoprotection in 20% sucrose solution until it sank, followed by immersion in 30% sucrose solution for 24 h. Coronal sections of 30 μm thickness were prepared using a cryostat microtome. After three PBS washes, the sections were blocked at room temperature for 2 h in blocking buffer (2% goat serum, 1% BSA, 0.1% Triton-X 100, 0.05% Tween-20 in PBS). The sections were incubated overnight at 4°C under constant agitation with primary antibodies against GR (Abcam; 1:500) or c-Fos (HUABIO; 1:500), diluted in primary antibody dilution buffer (3% BSA in PBS). The following day, after removing the primary antibodies, the sections were washed three times with PBS (10 min each), then incubated for 1 h at room temperature with fluorescently conjugated secondary antibodies (goat anti-rabbit IgG; Abcam; 1:1,000) diluted in PBS containing 3% BSA. After discarding the secondary antibodies and performing three additional PBS washes (10 min each), the sections were mounted using an antifade mounting medium containing DAPI. The target hippocampal CA1 region was localized according to the mouse brain atlas, imaged using a Nikon A1R confocal microscope, and quantitatively analyzed for GR and c-Fos fluorescence intensity using ImageJ software.

### 4.11 Western blot

Before the experiment, the mice were anesthetized with 0.7% pentobarbital sodium at 22:00–24:00 on day 21, and then euthanized by cervical decapitation. The brain tissue was promptly extracted from the euthanized mice and placed in a cryostat for freezing. After complete tissue freezing, the hippocampus was sectioned using the cryostat, and samples were obtained through microdrill drilling. Hippocampal samples were homogenized by sonication and then centrifuged to collect the supernatant. Equivalent amounts of protein were separated on 12.5% SDS–PAGE and transferred to PVDF membranes. Membranes were rinsed with TBST and then blocked in 5% nonfat milk for 1 h. Subsequently, overnight incubation was performed with primary antibodies diluted at 1:1,000 (endogenous control: GAPDH, β-actin; Solarbio China; primary antibodies: GR, Abcam, United States; FKBP51, Affinity, China; MR, SinoBiological, China). After washing with TBST, membranes were incubated for 1 h with HRP-conjugated secondary antibodies (goat anti-rabbit or goat anti-mouse; JINGKEBIO, China) diluted 1:2,000, followed by three additional TBST washes. Immunoreactive bands were developed using the Super ECL substrate (Shanghai Zeha Biotechnology Co., Ltd.) and imaged with a Bio-Rad gel documentation system. Band intensity (grayscale) was quantified using ImageJ (win64, v1.51).

### 4.12 Serum hormone test

Serum collection was performed between 22:00 and 24:00 on the 21st, which was scheduled after the completion of modeling and behavioral testing, mice were anesthetized by intraperitoneal injection of 0.7% sodium pentobarbital, and blood was collected via cardiac puncture. Samples were stored at 4°C overnight, then centrifuged at 4,000 rpm for 10 min to separate serum. Serum levels of CRH, CORT, ACTH, and AD were quantified using mouse ELISA kits (Sankang Biotechnology Co., Ltd.) according to the manufacturer’s protocols.

### 4.13 Brain tissue hormone test

From 22:00 to 24:00 on the 21st day, mice were deeply anesthetized by intraperitoneal injection of 0.7% sodium pentobarbital, and the brains were quickly removed. Tissues were embedded in a cryo-embedding medium and equilibrated at −20 °C for 30 min. Coronal cryosections containing the hippocampus, hypothalamus, and pituitary were prepared using a cryostat, after which the regions of interest were isolated with a tissue micro-punch and stored at −80 °C until analysis. For ACTH determination, pituitary samples from two mice per group were pooled, whereas hippocampal CORT and whole hypothalamic CRH were measured individually without pooling. Endogenous CORT, CRH, and ACTH levels were quantified using mouse ELISA kits (Hebei Xiankang Biology Co., Ltd., China) according to the manufacturer’s instructions.

### 4.14 Hematoxylin and eosin staining

Brain tissues were collected from mice 21 days after dual-frequency electromagnetic radiation exposure. The excised brains were fixed in 4% paraformaldehyde solution (tissue volume to fixative ratio = 1:10) at 4°C under light-protected conditions for 24–48 h. Subsequently, tissues were dehydrated through a graded ethanol series (70, 80, 95%, and twice in absolute ethanol; 1.5 h per step), cleared in xylene, and embedded in paraffin. Coronal sections of 5 μm thickness were serially cut, with target regions containing the hippocampal-hypothalamic complex precisely selected according to mouse brain stereotaxic coordinates (Bregma −1.5 to −2.5 mm).

Prior to staining, sections were dewaxed in xylene (three changes, 10 min each) and rehydrated through a reverse ethanol gradient (100% → 70%), followed by distilled water rinsing. Then tissues underwent nuclear staining by immersion in Harris hematoxylin containing 0.25% mercuric oxide for 8 min. This was followed by a 10-min wash under running water. Sections were then differentiated in 1% acid-alcohol for 5 s and immediately transferred to 0.2% ammonia water for bluing, lasting 30 s. Subsequently, cytoplasmic counterstaining was performed using a 0.5% eosin Y ethanol solution (containing 0.1% glacial acetic acid) for 1 min. Dehydration was then carried out rapidly through a graded ethanol series: 95% ethanol for 10 s, another bath of 95% ethanol for 30 s, and two changes of 100% ethanol for 1 min each. Clearing was achieved through three changes of xylene, each lasting 5 min. Finally, sections were mounted and cover slipped using neutral resin. Stained sections were imaged using a Nikon Eclipse Ci microscope equipped with a DS-Fi3 CCD camera at 200 × and 400 × magnification. All image data were subjected to quantitative analysis using ImageJ software.

### 4.15 RNA sequencing

Upon attaining complete anesthesia, the mice were dissected. From each group of nine independent experimental mice, three mice from the CA1 region of the hippocampus were pooled for transcriptome analysis. RNA sequencing was conducted by Novogene Co., Ltd. (Beijing, China). Differentially expressed genes (DEGs) were identified using an adjusted *p* < 0.05. Detailed experimental protocols are presented in Supplementary Information.

### 4.16 Stereotaxic surgery

Mice were anesthetized using 5% isoflurane (induction) and maintained at 2% isoflurane via a nose cone while secured in a stereotaxic frame (Kopf Instruments, Tujunga, CA). Ocular lubricant was applied to prevent corneal desiccation. Following a midline scalp incision, the skull surface was sequentially cleansed with 3% hydrogen peroxide and saline to ensure clear visualization of cranial landmarks. The skull was leveled such that the height differential between bregma and lambda did not exceed ± 0.05 mm, with lambda positioned slightly higher than bregma. Bilateral injections targeting the hippocampal CA1 region (stereotaxic coordinates relative to bregma: AP −2.0 mm, ML ± 1.80 mm, DV −1.80 mm ventral to dura) were performed using a 10 μL microsyringe (Hamilton Co., Reno, NV) mounted on a microprocessor-controlled micropump (World Precision Instruments, Sarasota, FL). Recombinant adeno-associated viruses (rAAV; OBiO Technology, Shanghai) were infused at 30 nL/min according to the following experimental groups per hemisphere:

Overexpression: pAAV-CMV-MCS-mCherry-tWPA (389 nL, 1.02 × 10^13^ vg/mL) or pAAV-CMV-Nr3c1-3 × FLAG-P2A-mCherry-tWPA (500 nL, 7.93 × 10^12^ vg/mL); Knockdown: pAAV-U6-shRNA (NC)-CMV-EGFP-WPRE (444 nL, 1.42 × 10^13^ vg/mL) or pAAV-U6-shRNA (Nr3c1)-CMV-EGFP-WPRE (500 nL, 1.26 × 10^13^ vg/mL).

The injection needle remained *in situ* for 10 min post-infusion to facilitate viral diffusion. The incision was subsequently sutured and disinfected. Animals received postoperative monitoring during recovery and were allowed 21 days for transgene expression prior to experimental assessments.

### 4.17 Statistical analysis

Statistical analyses were carried out in GraphPad Prism (v8.4.2) after assessing data normality. For comparisons between two groups, unpaired Student’s *t*-tests were applied, whereas one-way ANOVA was used when more than two groups were analyzed, Two-way ANOVA was employed for repeated measurements at multiple time points. Results are presented as mean ± SEM. Significance levels were defined as *p < 0.05, **p < 0.01, ***p < 0.001, and ****p < 0.0001.

## 5 Conclusion

**FIGURE 7:**
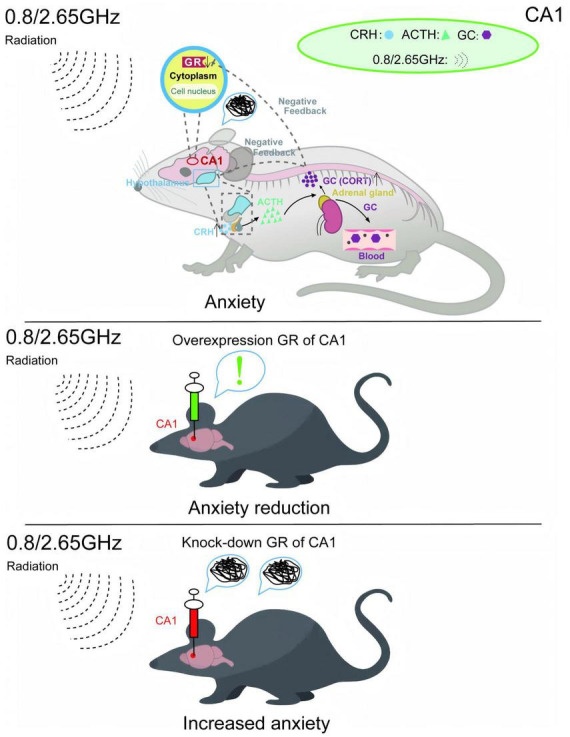
Influence of dual-frequency EMR (0.8/2.65 GHz) on the HPA axis and glucocorticoid receptor in the CA1. Anxiety-like behaviors observed in mice following exposure to dual-frequency EMR (0.8/2.65 GHz) were strongly associated with hyperactivation of the hypothalamic–pituitary–adrenal (HPA) axis, coupled with reduced GR levels in the CA1 region. Consistent with this, targeted overexpression of GR in the CA1 region markedly alleviated anxiety-related behaviors, whereas GR knockdown exacerbated them. CA1: Cornu Ammonis1. GR: glucocorticoid receptors. CRH: corticotropin releasing hormone. ACTH: adrenocorticotropic hormone. GC: glucocorticoid receptor (mainly composed of corticosterone).

In summary, this study demonstrates that anxiety-like behavior induced by dual-frequency EMR is mediated by dysregulation of the HPA axis and impaired GR function in the CA1 hippocampal region. Furthermore, overexpression of GR in the CA1 significantly alleviated anxiety-like behaviors, whereas knockdown of GR exacerbated these negative emotional responses (Figure 7). These findings provide mechanistic insight into EMR-induced anxiety and suggest promising avenues for therapeutic intervention.

## Data Availability

The datasets presented in this study can be found in online repositories. The names of the repository/repositories and accession number(s) can be found in the article/Supplementary material.
